# RT-PCR negative COVID-19

**DOI:** 10.1186/s12879-022-07095-x

**Published:** 2022-02-13

**Authors:** Heta Parmar, Margaret Montovano, Padmapriya Banada, Sri Ram Pentakota, Stephanie Shiau, Zhongjie Ma, Kaheerman Saibire, Abby Chopoorian, Michael O’Shaughnessy, Mitchell Hirsch, Priyanshi Jain, Gaiane Demirdjian, Magali Karagueuzian, Thomas Robin, Michael Salvati, Bhavana Patel, David Alland, Yingda L. Xie

**Affiliations:** 1 The Public Health Research Institute and the Department of Medicine, Rutgers New Jersey Medical School, Newark, NJ USA; 2 School of Medicine, Rutgers New Jersey Medical School, Newark, NJ USA; 3 Department of Biostatistics & Epidemiology, Rutgers School of Public Health, Piscataway, NJ USA; 4 Beckman Coulter Inc., Brea, CA USA; 5 University Hospital, Newark, NJ USA

**Keywords:** COVID-19, SARS-CoV-2, Suspects, RT-PCR, Diagnosis

## Abstract

**Background:**

COVID-19 is a multi-system infection with emerging evidence-based antiviral and anti-inflammatory therapies to improve disease prognosis. However, a subset of patients with COVID-19 signs and symptoms have repeatedly negative RT-PCR tests, leading to treatment hesitancy. We used comparative serology early in the COVID-19 pandemic when background seroprevalence was low to estimate the likelihood of COVID-19 infection among RT-PCR negative patients with clinical signs and/or symptoms compatible with COVID-19.

**Methods:**

Between April and October 2020, we conducted serologic testing of patients with (i) signs and symptoms of COVID-19 who were repeatedly negative by RT-PCR (‘Probables’; N = 20), (ii) signs and symptoms of COVID-19 but with a potential alternative diagnosis (‘Suspects’; N = 15), (iii) no signs and symptoms of COVID-19 (‘Non-suspects’; N = 43), (iv) RT-PCR confirmed COVID-19 patients (N = 40), and (v) pre-pandemic samples (N = 55).

**Results:**

Probables had similar seropositivity and levels of IgG and IgM antibodies as propensity-score matched RT-PCR confirmed COVID-19 patients (60.0% vs 80.0% for IgG, p-value = 0.13; 50.0% vs 72.5% for IgM, p-value = 0.10), but multi-fold higher seropositivity rates than Suspects and matched Non-suspects (60.0% vs 13.3% and 11.6% for IgG; 50.0% vs 0% and 4.7% for IgM respectively; p-values < 0.01). However, Probables were half as likely to receive COVID-19 treatment than the RT-PCR confirmed COVID-19 patients with similar disease severity.

**Conclusions:**

Findings from this study indicate a high likelihood of acute COVID-19 among RT-PCR negative with typical signs/symptoms, but a common omission of COVID-19 therapies among these patients. Clinically diagnosed COVID-19, independent of RT-PCR positivity, thus has a potential vital role in guiding treatment decisions.

**Supplementary Information:**

The online version contains supplementary material available at 10.1186/s12879-022-07095-x.

## Introduction

COVID-19 (‘COVID’), a multi-system infection caused by the novel coronavirus SARS-CoV-2, can manifest along a clinical spectrum from asymptomatic and mild upper respiratory infections to severe pneumonia with respiratory and multi-organ failure [1–3]. Despite the progress in discovery and validation of effective therapeutic approaches across different disease stages, clinical care systems have remained vulnerable to COVID surges. Effective diagnostic approaches to ensure comprehensive detection and appropriate treatment of COVID are critical to reducing COVID related morbidity and mortality.

Reverse transcriptase polymerase chain reaction (RT-PCR) is currently the primary test for diagnosis of acute COVID. The FDA issued emergency use authorizations to commercial RT-PCR tests based on test performance with known positive material from a patient or contrived upper respiratory specimens [4]. Use of either known or contrived samples can often result in overestimation of true clinical test sensitivity, since upper respiratory swabs may miss infected material in practice [5, 6]. The decline in SARS-CoV-2 shedding from upper respiratory tract specimens within the 1st week after symptom onset is associated with an increase in RT-PCR false negative rate of 2–29% [7–10]. Although RT-PCR has a higher positivity rate among lower respiratory specimens, most patients do not spontaneously produce sputum and would require potentially symptom-exacerbating or invasive measures to obtain lower respiratory tract samples [11–13]. Thus, a COVID diagnosis may be missed in hospitalized patients who often present further along in their infection, leading to delayed or missed intervention and unnecessary empiric therapies directed towards an inaccurate alternative diagnosis. While serologic tests have limited standalone value in diagnosing acute COVID at the individual level [14], we reasoned that comparing relative seropositivity rates early in the pandemic with COVID-confirmed and COVID-negative cohorts could affirm the likelihood of acute COVID in RT-PCR negative patients with clinical signs/symptoms of COVID. This practice was aided by the timing of our study, which was performed April–October 2020, prior to the roll-out of vaccines and when the statewide seroprevalence in New Jersey was relatively low (11.9–14.8% between July and Oct 2020) [15, 16].

## Methods

### Participants

This study was approved by Rutgers University Institutional Review Board (Pro2020000861) and conducted at University Hospital (UH) in Newark, NJ, which had adopted early guidelines to screen all patients by SARS-CoV-2 RT-PCR. UH patients were screened 1–2 times per week by an Infectious Disease physician between April and October 2020 using the electronic medical records (EMR) for one of four cohorts: (1) PCR-confirmed COVID-19 (‘PCR-confirmed’), (2) COVID Probable (‘Probables’), (3) COVID Suspects (‘Suspects’), and (4) COVID Non-suspects (‘Non-suspects’) [17]. Specifically, Probables and Suspects were identified by running an EMR report for all UH patients with at least 2 negative RT-PCR tests or referred by a provider based on COVID suspicion, who were in the hospital or emergency room within the past 6 days, then performing a chart review of patients who had not been previously screened. Because all UH patients were tested for COVID by RT-PCR, multiple rather than single negative RT-PCR tests were queried with the rationale that presence of clinical suspicion led providers to repeat the RT-PCR test. Patients with chest radiographic findings compatible with COVID pneumonia (multifocal, atypical, and/or viral pneumonia as assessed by a trained radiologist) or at least 3 of the following: acute to subacute onset of fevers or chills, cough, shortness of breath, hypoxia, anosmia/aguesia, altered mental status, sore throat, diarrhea/nausea/vomiting, headaches, myalgias/generalized weakness, or COVID exposure (known or suspected) were considered Probables if there was no clear alternative diagnosis. On the other hand, patients were considered Suspects if they met the above clinical criteria but had a potential alternative diagnosis documented by the care-taking provider (e.g. pulmonary edema, bacterial pneumonia) yet COVID could not be entirely ruled out as a competing or additional process. PCR-confirmed patients were identified by running a report for hospitalized patients with at least one positive SARS-CoV-2 RT-PCR test result and were in the hospital or emergency room within the last 6 days. Because any leftover blood was only available for the study within a 4–6 day window after collection from the patient, patients were only considered for PCR-confirmed, Probables, Suspects, or Non-suspects cohorts if they had blood collected for hematology labs in the past 4–6 days. Patients were excluded if no leftover blood was available in the hematology lab. To allow for comparability of serostatus between PCR-confirmed and Probables, the larger pool of identified PCR-confirmed patients were propensity score matched to the Probables in a 2:1 ratio by age, sex, symptom duration, and disease severity on the day of blood collection. Disease severity was defined as: asymptomatic (no documented COVID-19 symptoms), mild-moderate (symptomatic but not hypoxic), hypoxic not requiring ICU care, and hypoxic requiring ICU care. On the other hand, Non-suspects were identified by selecting patients in September and October 2020 with at least one negative SARS-CoV-2 RT-PCR without clinical signs/symptoms associated with COVID (e.g. trauma, psychiatric admissions). Non-suspects were also selected to match by age, sex, body mass index (BMI), and co-morbidities to the Probables group. Selection of patients were blinded to SARS-CoV-2 serologic status, which was not routinely performed at UH. Finally, we included existing serologic data from 55 pre-pandemic controls whose blood samples were collected before 2019 as part of routine clinical diagnosis (provided by Beckman Coulter and BioIVT, Westbury, NY).

### Sample and data collection

For the study, blood was collected in accordance to routine UH hematology lab procedure (Additional file 2: Additional methods). Additionally, clinical information was collected from the EMR including medical history, symptoms, disease severity, laboratory and radiological findings, and any agents given for the indication of treating COVID (e.g. dexamethasone, convalescent plasma, tocilizumab, remdesivir, and hydroxychloroquine at the outset of the pandemic) during the hospitalization. Time of infection onset of asymptomatic cases was estimated from symptomatic patients with mild disease severity.

### IgG and IgM serologic assay

To evaluate whether Probable and Suspects are truly COVID-19 positive, plasma samples from patients in all cohorts were analyzed for IgG and IgM antibodies. Undiluted plasma samples were heat inactivated for 30 min at 56 °C as per the institutional IBC protocol and tested using the FDA EUA-approved Access SARS-COV-2 assays (Beckman Coulter, Brea, CA, USA) [18] that detect Human IgM and IgG against the receptor-binding domain (RBD) of the SARS-CoV-2 S1 protein (Additional file 2: Additional methods).

### Surrogate neutralization assay

To validate our findings on a separate serologic assay with additional estimation of neutralizing antibody titers, we evaluated all cohorts using the SARS-CoV-2 surrogate neutralization test kit (Genscript, Piscataway NJ) which measures percentage of inhibition of binding of RBD to recombinant human ACE2, as per manufacturer’s instructions [19]. Inhibition percentage ≥ 30% was considered positive for neutralizing antibodies (Additional file 2: Additional methods).

### Statistical methods and sample size considerations

Prior to data analysis, a pre-determined target sample size for Probable and Non-suspects was estimated by Fisher’s Exact Test for difference of Proportion, using SAS 9.4 (SAS Institute, Cary NC). Sample size calculations were driven by the following assumptions: (a) Seropositivity rate among Non-suspects of 15% was assumed to be similar to the background seroprevalence at the time; (b) seropostivity rate among Probables would always be higher than among Non-suspects; (c) seropositivity rate among Probables was estimated to be > 55% (background seroprevalence + 40% seroconversion rate due to acute COVID); (d) power ≥ 80%; (e) alpha of 0.05; and (f) ratio of Non-suspects to Probables would be 2:1 given a larger recruitment pool of Non-suspects. With these assumptions as inputs we computed the required sample size for a One-sided Fisher’s Exact Conditional Test for Two Proportions. Per our calculation a total of 48 study patients (32 Non-suspects: 16 Probables) would be required to detect a 40% excess seropositivity in the Probables. In our study we oversampled and enrolled 43 Non-suspects and 20 Probables with an actual power of 89%.

All categorical data were described using numbers and percentage, and comparisons were made between groups using Fisher’s exact test. All continuous data were described as median [25th–75th percentile] and comparisons between groups were made using non-parametric two-sample Wilcoxon Signed Rank test. Statistical analyses were primarily conducted using R and Graph-Pad Prism version 9. Propensity score matching of PCR-confirmed to Probables was performed with PSMATCH procedure using SAS 9.4.

## Results

### Clinical characteristics

**Fig. 1:**
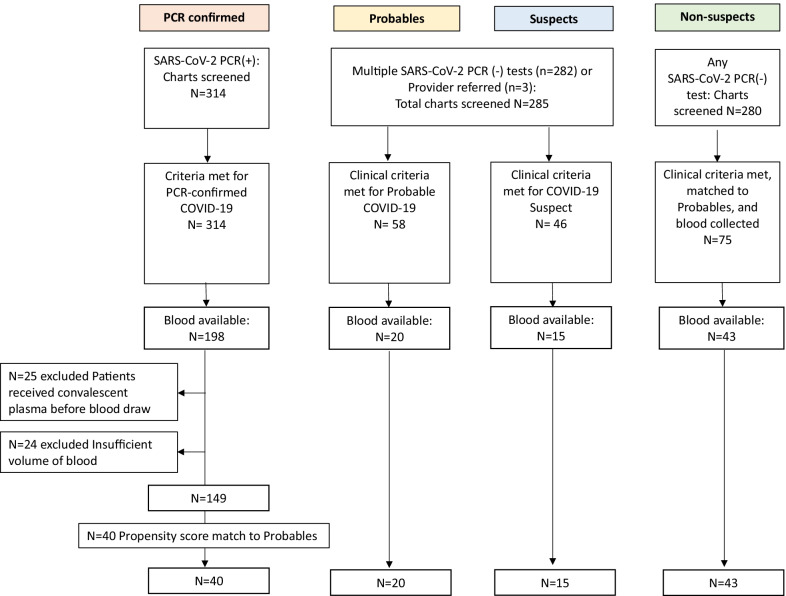
Flowchart of patients and samples included in the analysis

We identified 314 PCR-confirmed, 58 Probables, 46 Suspects, and 75 Non-suspects who met clinical criteria, of whom leftover blood was available for 198 (63.1%), 20 (34.5%), 15 (32.6%), and 43 (57.3%), respectively (Fig. 1). The 20 Probables and 15 Suspects with available blood were identified from a review of 285 charts belonging to patients with more than one SARS-CoV-2 negative PCR test during the study period (N = 282) or referred by a provider based on COVID suspicion (N = 3) (Fig. 1). Of the 198 PCR-confirmed patients with blood available in the hematology lab, 49 were excluded as they received convalescent plasma before blood draw or due to insufficient volume of blood. From the remaining 149 PCR-confirmed patients, 40 PCR-confirmed (twice the number of Probables) were selected by propensity score matching to Probables. Finally for the Non-suspects, review of a total of 280 charts of patients with any negative SARS-CoV-2 PCR test identified 75 patients without COVID signs or symptoms who matched to the Probables group, and had blood collected in the previous 4–6 days. Among these 75 patients, 43 had leftover blood available in the hematology lab for the study. Therefore, altogether, 40 PCR-confirmed, 20 Probables, 15 Suspects, and 43 Non-suspects were included in the analysis.

Overall, the PCR-confirmed, Probables, and Non-suspects were balanced in baseline clinical characteristics that were matched between the PCR-confirmed vs Probables, and Probables vs Non-suspects groups (Table 1). Age, BMI, sex, and race/ethnicity were not significantly different between these four cohorts. As expected, the distribution of disease severity of PCR-confirmed and Probable patients on day of blood collection for serologic assessments, ranging from mild to critical, was also similar by matching.

**Table 1:** Clinical characteristics of all patients in four cohorts:

	N	COVID-19 patient cohorts				P1	P2	P3
		PCR confirmed	Probables	Suspects	Non-Suspects			
		40	20	15	43			
Median (IQR)	Age (years)	60.0 (43.5–65.3)	53.0 (40.5–64.5)	57.0 (47.5–62.5)	53.0 (46.5–61.0)	0.742	0.740	0.941
Median (IQR)	BMI	26.4 (24.0–32.5)	30.8 (26.0–35.0)	25.9 (24.2–32.9)	28.1 (25.0–31.4)	0.101	0.217	0.111
N (%)	Male	28 (70.0%)	13 (65.0%)	8 (53.3%)	31 (72.1%)	0.772	0.510	0.570
Median (IQR)	Days between symptom onset and RT-PCR test	6 (4–7)	3 (1–9)	1 (0–5)	NA	0.676	0.154	NA
Median (IQR)	Days between symptom onset and Ab test	11 (7–17)	14 (4–24)	14 (4–16)	NA	0.655	0.688	NA
	Race/Ethnicity							
N (%)	Black or African American	19 (47.5%)	8 (40.0%)	8 (53.3%)	26 (60.4%)	0.735	0.809	0.318
N (%)	Hispanic or Latino	13 (32.5%)	9 (45.0%)	5 (33.3%)	12 (28.0%)			
N (%)	Caucasian	6 (15.0%)	3 (15.0%)	2 (13.3%)	4 (9.3%)			
N (%)	Others	2 (5.0%)	0 (0.0%)	0 (0.0%)	1 (2.3%)			
N (%)	Chest imaging findings consistent with COVID	26 (65.0%)	18 (90.0%)	13 (86.7%)	NA	0.061	1.00	NA
N (%)	Symptoms and risk factors							
N (%)	Fever	14 (35.0%)	8 (40.0%)	3 (20.0%)	0 (0.0%)	0.779	0.281	< 0.001
N (%)	Coughing	12 (30.0%)	8 (40.0%)	5 (33.0%)	0 (0.0%)	0.563	0.737	0.002
N (%)	Dyspnea	19 (47.5%)	14 (70.0%)	11 (73.3%)	0 (0.0%)	0.168	1.00	< 0.001
N (%)	Chills	6 (15.0%)	4 (20.0%)	1 (6.7%)	0 (0.0%)	0.718	0.365	0.008
N (%)	Sore throat	1 (2.5%)	1 (5.0%)	0 (0.0%)	0 (0.0%)	1.000	1.000	0.317
N (%)	Diarrhea	4 (10.0%)	4 (20.0%)	2 (13.3%)	0 (0.0%)	0.421	0.680	0.008
N (%)	Altered mental status	3 (7.5%)	2 (10.0%)	1 (6.7%)	0 (0.0%)	1.000	1.000	0.090
N (%)	Known or suspected exposure	2 (5.0%)	1 (5.0%)	0 (0.0%)	0 (0.0%)	1.000	1.000	NA
	Disease severity at admission							
N (%)	Asymptomatic	10 (25.0%)	2 (10.0%)	*3 (20.0%)	NA	0.264	0.607	NA
N (%)	Mild-moderate	11 (27.5%)	4 (20.0%)	6 (40.0%)	NA			
N (%)	Hypoxic-no ICU	18 (45.0%)	12 (60.0%)	5 (33.3%)	NA			
N (%)	Critical-ICU	1 (2.5%)	2 (10.0%)	1 (6.7%)	NA			
	Disease severity at collection							
N (%)	Asymptomatic	8 (20.0%)	0 (0.0%)	*3 (20.0%)	NA	0.079	0.151	NA
N (%)	Mild-moderate	14 (35.0%)	12 (60.0%)	7 (46.7%)	NA			
N (%)	Hypoxic-no ICU	16 (40.0%)	7 (35.0%)	3 (20.0%)	NA			
N (%)	Critical-ICU	2 (5.0%)	1 (5.0%)	2 (13.3%)	NA			
	Disease severity at peak							
N (%)	Asymptomatic	8 (20.0%)	0 (0.0%)	*3 (20.0%)	NA	0.039	0.209	NA
N (%)	Mild-moderate	8 (20.0%)	4 (20.0%)	4 (26.7%)	NA			
N (%)	Hypoxic-no ICU	22 (55.0%)	12 (60.0%)	6 (40.0%)	NA			
N (%)	Critical-ICU	2 (5.0%)	4 (20.0%)	2 (13.3%)	NA			
	Chronic medical conditions							
N (%)	Hypertension	21 (52.5%)	11 (55.0%)	7 (46.7%)	25 (58.1%)	1.000	0.738	1.000
N (%)	Heart disease	5 (12.5%)	3 (15.0%)	4 (26.7%)	10 (23.2%)	1.000	0.430	0.520
N (%)	Diabetes	13 (32.5%)	7 (35.0%)	5 (33.3%)	10 (23.2%)	1.000	1.000	0.370
N (%)	Liver disease	2 (5.0%)	3 (15.0%)	6 (40.0%)	2 (4.7%)	0.322	0.129	0.315
N (%)	Lung disease	7 (17.5%)	6 (30.0%)	5 (33.3%)	5 (11.6%)	0.326	1.000	0.060
N (%)	Kidney disease	6 (15.0%)	4 (20.0%)	5 (33.3%)	7 (16.3%)	1.000	0.246	1.000
	Microbiology							
N (%)	Positive respiratory pathogen panel	0 (0.0%)	1 (5.0%)	0 (0.0%)	NA	0.154	1.000	NA
N (%)	Positive sputum culture	4 (10.0%)	4 (20.0%)	2 (13.3%)	NA	0.283	0.680	
N (%)	Positive blood culture	5 (12.5%)	2 (10.0%)	2 (13.3%)	NA	0.776	1.000	
N (%)	Positive urine culture	8 (20.0%)	6 (30.0%)	6 (40.0%)	NA	0.388	0.721	
	Treatments^#^							
N (%)	COVID directed (any)	23 (71.8%)	7 (35.0%)	2 (16.7%)	0	0.008	0.264	NA
	Length hospital days							
Median (IQR)		14 (7.8–19.3)	16 (7.0–23.0)	15 (5.5–22.5)	NA	0.813	0.676	NA
	Outcomes							
N (%)	Uninfected	NA	NA	NA	43 (100.0%)			NA
N (%)	Survived	36 (90.0%)	19 (95.0%)	14 (93.3%)	NA	0.656	1.000	

P1 = p-value between Probables and PCR-confirmed, P2 = p-value between Probables and Suspects, P3 = p-value between Probables and Non-suspects

*NA* not available; *IQR* inter quantile range; *PCR* polymerase chain reaction, *ICU* Intensive Care Unit; *BMI* Basal Metabolic Index

*3 Suspects were asymptomatic (no documented COVID-19 symptoms) but had chest radiographic findings read as “viral/multifocal pneumonia” or “viral infection”

^#^Denominator includes only symptomatic patients at collection (N = 32 PCR-confirmed, N = 20 Probables, N = 12 Suspects)

**Fig. 2:**
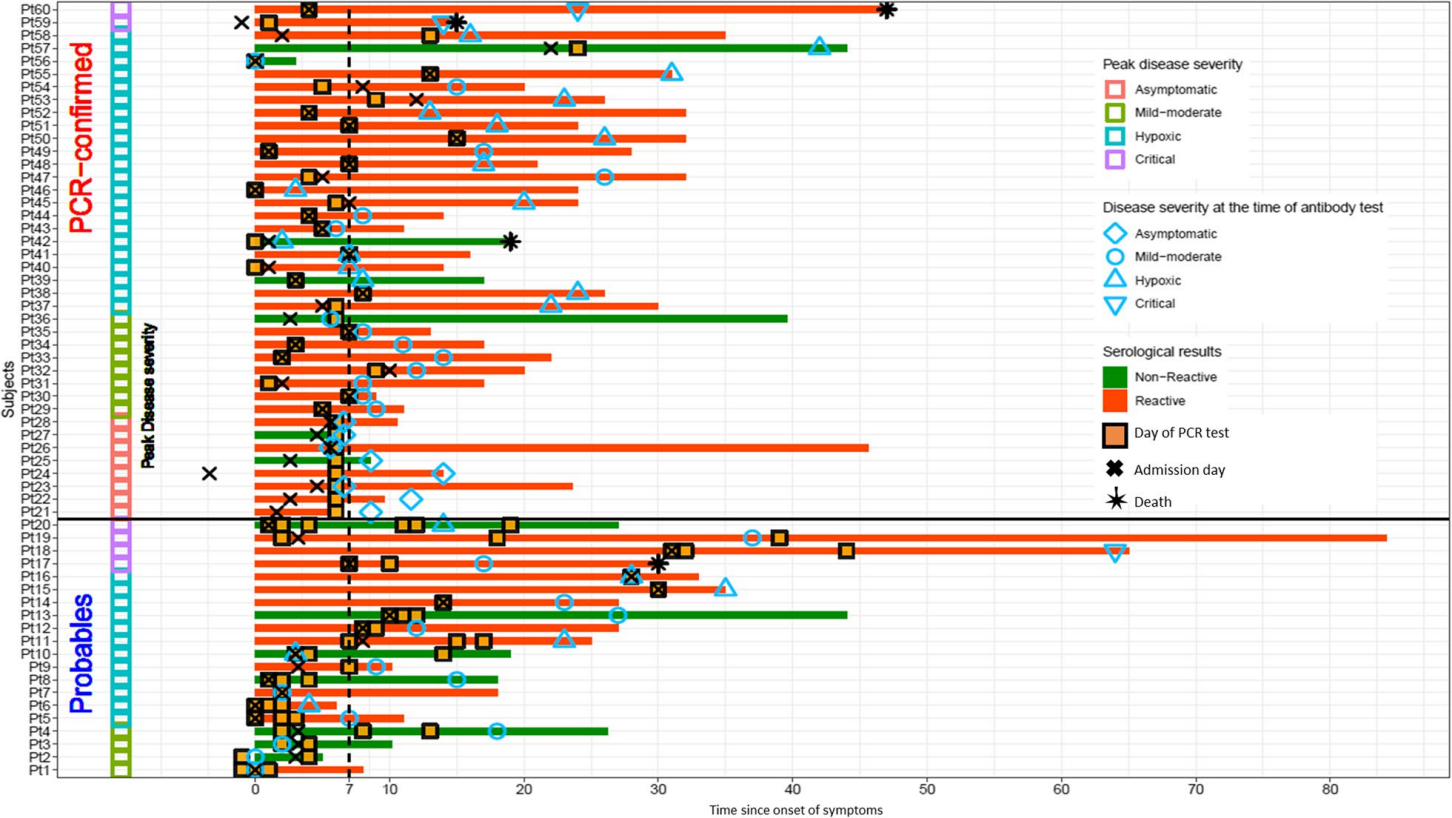
Swim plot with *x*-axis illustrating time since onset of symptoms in days and *y*-axis represents patients. The day of hospital admission represented by black crosses, RT-PCR test timeline by orange squares, disease severity at the time of antibody test by blue diamond = asymptomatic, blue circle = mild-moderate, blue triangle = hypoxic-NO ICU, blue inverse triangle = critical. The Probables (Pt 1–20, N = 20) and matched PCR-confirmed (Pt 21–60; N = 40). Sample with (red bars = Reactive) and without (green bars = Non-reactive) COVID-19 specific antibodies at any point of blood draw. Open squares indicates peak disease severity and black star indicates deceased state at end of hospitalization. A small number of patients (Pt 11, 12, 24, 59) were PCR tested for prior hospitalization as part of routine screening while few patients developed symptoms after hospitalization. Pt 7, 15 and 16 were provider referred based on meeting clinical criteria, while Pt 9 and 14 had two RT-PCR tests carried out at different times on the same day

The most common symptoms among Probables and PCR-confirmed were dyspnea, followed by fevers and cough, and followed by chills and diarrhea (Table 1). In contrast, most common symptoms among Non-suspects were pain of extremities, back or abdomen in the setting of trauma, fall, cirrhosis or pancreatitis. Probables were more likely to have chest imaging consistent with COVID compared to Suspects or PCR-confirmed, as this was a clinical sign used to identify Probables. Potential alternative diagnoses for Suspects were commonly heart failure exacerbation, bacterial pneumonia, and COPD exacerbation. All patients were tested for SARS-CoV-2 infection by RT-PCR at or soon after admission. The median time of the first RT-PCR test from symptom onset was not significantly different between PCR-confirmed (6 days [IQR 3.8–7.0]) and Probables (2.5 days [IQR 1.0–8.5]), whereas RT-PCR was performed earlier for Suspects (1 day [IQR 0.0–4.5]). Suspects and Probables had a median of 2 negative RT-PCR tests. Fourteen of 20 (70.0%) Probables and 33/40 (82.5%) of PCR-confirmed were first tested within the 7-day period when the PCR is most likely to be positive [20] (Fig. 2).

### IgG and IgM immunoassays

**Fig. 3:**
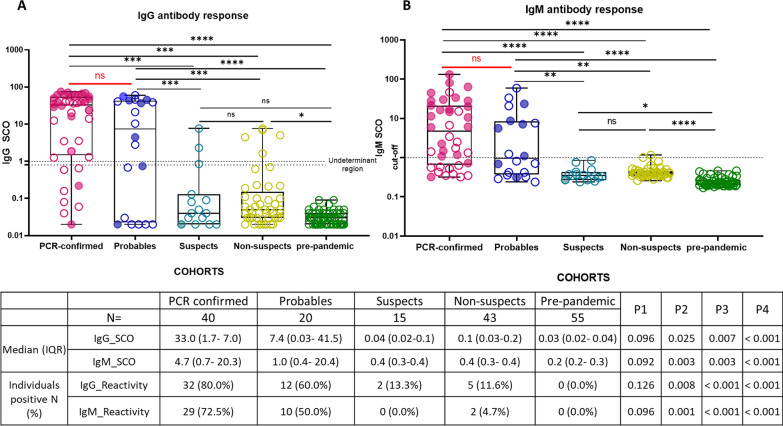
Signal to cut-off ratio of **A** IgG and **B** IgM among pre-pandemic samples collected before 2019, RT-PCR and no clinical suspicion of COVID-19 (Non suspects), RT-PCR negative with clinical signs/symptoms of COVID-19, with no potential alternate diagnosis (Probables) and with alternate diagnosis (Suspects) and matched RT-PCR confirmed for COVID-19. Boxplot indicates the interquartile range as the box and the minimum and maximum values as whiskers. Dashed line indicates cut-off values for call for reactivity. Filled circles are individuals who received COVID-19 directed therapies. Comparison between groups were by two-sided Wilcoxon signed rank test. P1 > p-value between PCR-confirmed and Probables, P2 > p-value between Probables and Suspects, P3 > p-value between Non-suspects and Probables and P4 > p-value between Pre-pandemic and Probables

The Access 2 assays demonstrated 100% specificity with no cross-reactivity from 55 plasma samples collected before end of 2019 (Fig. 3). Between PCR-confirmed and Probables, there was no difference in the IgG and IgM seropositivity rates (80.0% vs 60.0% for IgG, p-value = 0.236; and 72.5% vs 50.0% for IgM, p-value = 0.096) or levels (median IgG SCO 34.0 vs 7.4, p-value = 0.096; and median IgM SCO 4.7 vs 1.0, p-value = 0.092, respectively). Probables had substantially higher seropositivity rates than Suspects and Non-suspects (60.0% vs 13.3% and 11.6% for IgG, p-value 0.008 and < 0.001; 50.0% vs 0.0% and 4.7% for IgM, p-value 0.001 and < 0.001, respectively; Fig. 3). There were no significant differences in seropositivity rates and levels between the Suspects, Non-suspects and Pre-pandemic cohorts.

### Surrogate neutralization studies

**Fig. 4:**
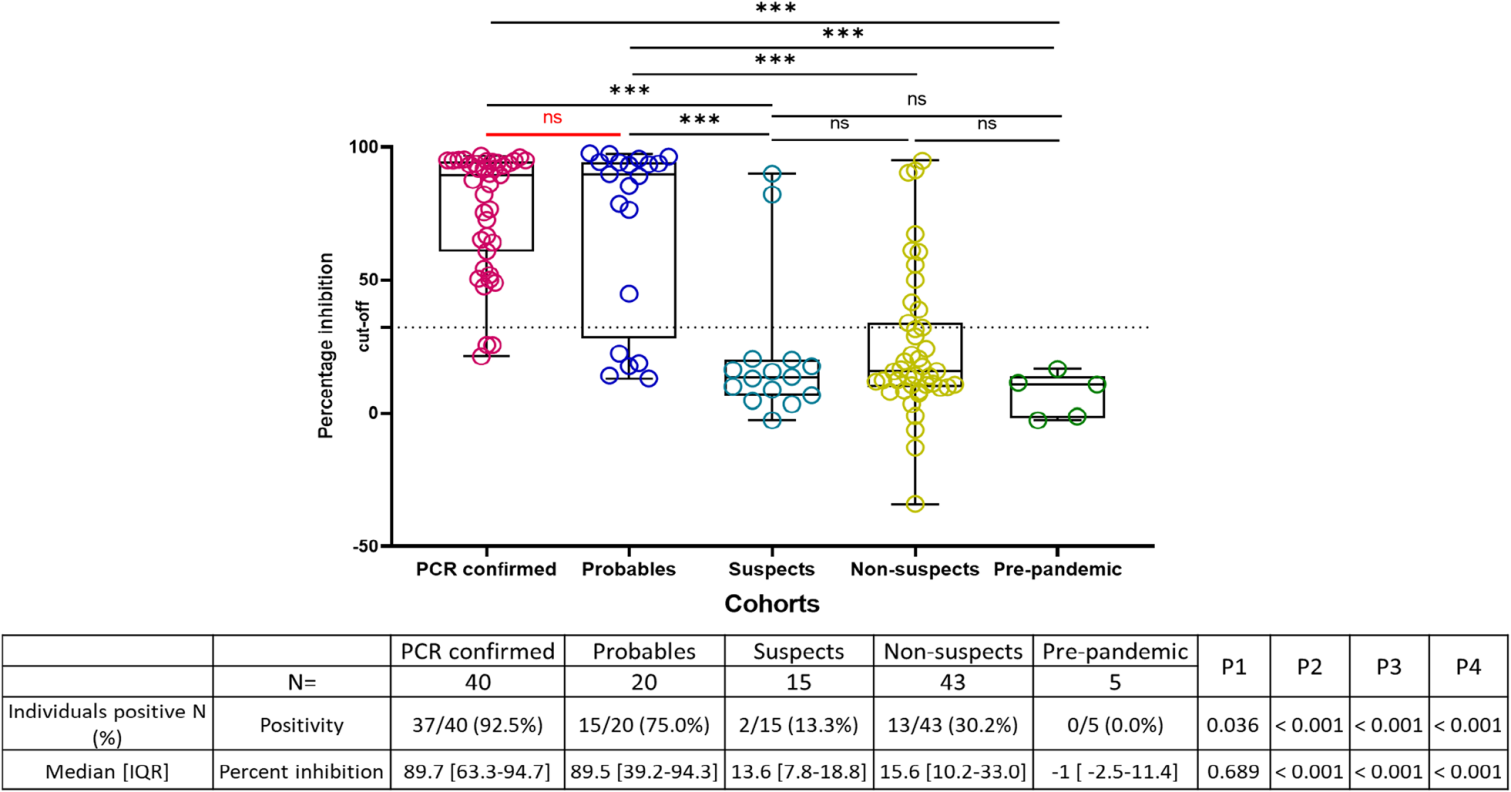
Neutralizing antibody response among Pre-pandemic, Non-suspects, Suspect, Probables and PCR-confirmed cohorts. Neutralization at the 1:20 dilution of plasma samples was measure of percent inhibition. The dashed line indicates percent inhibition cut-off. Comparison between groups were by two-sided Wilcoxon signed rank test. P1 > p-value between PCR-confirmed and Probables, P2 > p-value between Probables and Suspects, P3 > p-value between Non-suspects and Probables and P4 > p-value between Pre-pandemic and Probables

PCR-confirmed had a higher neutralization rate than Probables (92.5% vs 75.0%, p-value = 0.036), although their median percent inhibition were similar (median 89.7% vs 89.5%, p-value = 0.689) (Fig. 4). Probables had a significantly higher neutralization rate and percent inhibition than Suspects, Non-suspects and pre-pandemic controls (Fig. 4).

### Seropositive versus seronegative Probables

Although the numbers were small, the 15 Probables with neutralizing antibodies were more likely to have a longer median duration of symptoms, more severe disease, and receive COVID-directed treatment compared to the Probables without neutralizing antibodies (Additional file 2: Table S2). There was a non-significant tendency for these differences when comparing IgM/IgG seropositive vs seronegative Probables (Additional file 2: Table S3). Otherwise, Probables with and without serologic evidence of SARS-CoV-2 could not be distinguished by age, BMI, race/ethnicity, or co-morbidities.

### Treatment initiation rates

During the study, new guidance was issued for dexamethasone and remdesivir as COVID-19 treatment for hospitalized patients at UH who require supplemental oxygen. Despite the similar rates of disease progression, only 35.0% of the symptomatic Probables received COVID treatment compared to 71.8% of the symptomatic PCR confirmed (p-value 0.008; Table 1). Among the 13 Probables that were positive by the IgG or IgM antibody tests, 6 (46%) received therapies for COVID-19. Among cases that required oxygen during their hospitalization (e.g. severe or critical peak disease severity), 7/16 (43.8%) Probables versus 19/24 (79.2%) PCR-confirmed received COVID-directed treatment (p-value = 0.017). Despite the differences in COVID-directed treatment, 19/20 (95.0%) of Probables and 36/40 (90.0%) of PCR-confirmed cases recovered from their illness and were discharged, irrespective of their disease severity and diagnosis (p = 0.66).

## Discussion

We identified a cohort of patients that was repeatedly RT-PCR negative for SARS-COV-2, despite presenting across a range of typical COVID associated signs and/or symptoms. In absence of an alternative diagnosis, these COVID-Probable patients had multi-fold higher IgG and IgM seropositivity and neutralization rates compared with background seroprevalence approximated by matched Non-suspect controls. Furthermore, the seropositivity, IgG and IgM levels, and neutralization potency among Probables were not significantly different than that of PCR-confirmed COVID patients who were propensity score matched by age, symptom duration, and disease severity. Although serology has limited value to diagnosis of acute COVID as we cannot rule out seroprevalence [21, 22] or pre-existing neutralizing antibodies among the Probables at an individual level [23, 24], their aggregate SARS-CoV-2 serologic profiles was comparable to that of PCR-confirmed COVID and multifold higher than background prevalence represented by Non-suspects. These results support a substantial likelihood that acute COVID-19 was present in our Probable group.

Importantly, despite similar seropositivity and disease severity rates, our PCR-confirmed cohort was twice as likely to receive COVID treatment than our Probables cohort. This discrepancy implicates missed potential opportunities to prevent early progression of mild-moderate disease in high-risk patients, and potentially reduce morbidity and/or mortality in severe disease. We incidentally observed that 43% of the Probables who presented with mild-moderate disease progressed to severe hypoxic disease, thus representing a window for interventions that reduce disease progression. Among the seropositive Probables who had or developed severe disease, only 37.5% received steroid therapy, which is associated with reduced mortality in severe disease [25]. Although the study was not powered to look for treatment outcomes, the consequences of missed treatment opportunities are likely to have growing costs with emergence of new effective treatment approaches. These observations support a heightened consideration of evidence-based COVID therapies in RT-PCR negative patients with COVID signs/symptoms and no obvious alternative diagnosis.

We additionally included a group of Suspects that shared signs and symptoms of COVID but with a potential alternative or concomitant diagnosis. Whereas Suspects fell across a spectrum of COVID suspicion, their level of clinical suspicion was lower than that of Probables. Interestingly, rather than falling on a spectrum between Probables and Non-suspects, we observed that the seropositivity and neutralization rates of these Suspects were not significantly different than those of Non-suspects. These findings suggest that the presence of a potential alternative diagnosis markedly reduces the likelihood of acute COVID while increasing the likelihood of capturing seroprevalence from prior exposure. Of note, while we kept the criteria for COVID signs and symptoms broad to capture the diverse range of COVID clinical manifestations, this increases the possibility of alternative diagnoses with overlapping signs and symptoms. The proportion of Suspects to Probables—and thus the likelihood of acute COVID using this clinical criteria will vary depending on the prevalence of COVID versus competing diagnoses of a population. For example, during our study period, other respiratory viruses that could cause a similar clinical presentation (e.g. common human coronaviruses, influenza, parainfluenza, HMPV) were low [26] but would be an important competing alternative diagnosis in settings where they are circulating.

The study had several limitations. The primary limitation is that the Probables in this study with available blood underestimated the total number of Probables during this period given the lack of a comprehensive method to screen for all individuals at UH with clinical signs and symptoms of COVID-19. The small sample size based on statistical estimates may not capture important variability across populations and centers of COVID Probables and Suspects. While expansion to larger, multicenter studies could increase confidence in the generalizability of the findings, the ability to reproduce this study now is diminished by high background seroprevalence rates in the present post-vaccination era. Secondly, the study was also not designed nor powered to describe the epidemiology or outcomes of RT-PCR negative COVID. Although we were not able to evaluate the prevalence of RT-PCR negative COVID-19 suspects in our patient population, their considerable prevalence has been suggested previously [27]. Thirdly, we had limited longitudinal serologic data to determine if some of the earlier seronegative Probables converted to seropositive. Due to duration of hospitalization or interval for collection of blood, some of the only time-points available were within 1 week of symptom onset. Therefore, we were not able to comment on the added value of serial serologic testing, which may have detected more suspects with early acute infection. However, in this comparative analysis, we attempted to control for seronegativity rates with early time-points by matching time from symptom onset to serologic collection between the PCR-confirmed and Probable groups. Even so, we did find in our study cohorts that 52% of all PCR-confirmed, Probables, and Suspects were seropositive within 1 week of symptom onset, corresponding with an important timeframe for diagnosis and early treatment initiation. Finally, the COVID-Non-suspects were, on average, identified at a later time interval than the Probables. However, this would bias towards our estimates being conservative—that is, the background seroprevalence was likely lower during the timeframe Probables were identified and thus the Probables seropositivity minus background seroprevalence is likely even higher than estimated in this study.

## Conclusions

This study identified a cohort of RT-PCR negative patients with clinical signs/symptoms of acute COVID with serologic evidence of SARS-CoV-2 infection that was multifold higher than Non-suspects and comparable to that of RT-PCR confirmed COVID. Despite similar (matched) disease severity, these RT-PCR negative patients were approximately half as likely to receive treatment as the RT-PCR confirmed COVID patients. Among the ongoing threat of highly-infectious variants and emergence of new evidence-based COVID therapies, these findings suggests a critical role for clinically-diagnosed COVID, whereby a negative RT-PCR test should not preclude evidence-based COVID treatment in the presence of clinical suspicion and no potential alternative diagnosis.

## Supplementary Information

**Additional file 1.** Study Dataset.

**Additional file 2.** Additional Methods and Results. **Table S1.** Clinical characteristics of PCR-confirmed or Probable cases with high neutralizing antibodies. **Table S2.** Clinical characteristics of Probables positive or negative of neutralizing antibodies. **Table S3.** Clinical characteristics of seropositive vs seronegative Probables for IgG or IgM.

## Data Availability

All data generated or analysed during this study are included in this published article and its Additional files 1, 2.
