# Supplementary material for: RT-PCR negative COVID-19
Source: BMC Infect Dis. 2022 Feb 13;22:149. doi: 10.1186/s12879-022-07095-x (PMC8841043; doi:10.1186/s12879-022-07095-x)
Supplement: Supplementary file 2 — Additional file 2. Additional Methods and Results. Table S1. Clinical characteristics of PCR-confirmed or Probable cases with high neutralizing antibodies. Table S2. Clinical characteristics of Probables positive or negative of neutralizing antibodies. Table S3. Clinical characteristics of seropositive vs seronegative Probables for IgG or IgM. [file 12879_2022_7095_MOESM2_ESM.docx]

**Additional Methods.**

Sample collection and processing: For the study available blood in K2EDTA-coated vacutainers was used for all subjects. In accordance with routine UH Hematology lab procedures, available leftover blood samples collected for the study were 4-6 days old and stored at 4°C. All blood samples were centrifuged; plasma was collected and stored at -80^o^C.

Immunological assay: Access SARS-COV-2 IgG and IgM are a two-step chemiluminescent-based enzyme immunoassays. Access SARS-COV-2 IgM assay captures Human IgM and utilizes a recombinant SARS-CoV-2 protein with alkaline phosphatase conjugate containing the receptor-binding domain of the viral S1 protein. Access SARS-COV-2 IgG assay detects human IgG and utilizes a monoclonal anti-human IgG alkaline phosphatase conjugate which binds to the IgG antibodies captured on the paramagnetic particles coated with recombinant protein from SARS-CoV-2. The light generated by the reaction of a chemiluminescent substrate and alkaline phosphatase (conjugated to the recombinant SARSCoV-2 protein in SARS-COV-2 IgM assay or conjugated to the monoclonal anti-human IgG in SARS-COV-2 IgG assay) is measured with a luminometer. The presence or absence of anti‐SARS‐CoV‐2 IgM or anti‐SARS‐CoV‐2 IgG in the patient sample is determined by the relative light intensity over the cut‐off value determined during calibration on the instrument. The Access system has a sensitivity of 92.7% (95% CI 88.1% - 95.6%) and specificity of 99.6% (95% CI 99.2%-99.9%) for IgG among 192 PCR-confirmed vs 1400 plasma and serum samples collected prior to December 2019 in France and United States. The IgM assay had a sensitivity of 91.3% (95% CI 86.2-94.7%)and specificity of 99.9% (95% CI 99.5-100% 173 PCR-confirmed vs 1400 plasma and serum samples collected prior to December 2019^1^.

Neutralization assay: We validated the serology results with SARS-CoV-2 surrogate neutralization assay with FDA EUA approval that detects total neutralizing antibodies to the SARS-CoV-2 receptor binding domain (RBD) as a measure of percent inhibition^2^. All samples and standards were prepared at a final dilution of 1:20 and performed as per manufacturer’s instruction. The test is a blocking ELISA detection assay, which mimics the virus neutralization process. The presence of neutralizing antibodies is indicated by decreases in absorbance produced by Horseradish peroxidase conjugated to RBD (RBD-HRP) as interaction between RBD-HRP and Human ACE2 receptor protein in blocked. Provided negative control must have optical density > 1.0 and positive control (contains neutralizing antibodies) must have optical density < 0.3. The inhibition percentage was calculated against provided negative controls. The assay has 100% clinical concordance with plaque reduction neutralization test ^3^.

**Additional Results.**

Surrogate neutralization studies: Percent inhibition correlated with IgG levels and symptom duration among PCR-confirmed than Probables (Figure S1). Just under half of PCR-confirmed and Probables had >90% inhibition. Comparing PCR-confirmed and Probables with >90% inhibition, Probables were younger (64 vs 46 years, p-value= 0.03), but did not significantly differ in disease severity (p=0.408) and days post symptom onset (p-value=0.257), yet COVID directed treatment was significantly biased (Table S1).

Figure S1: Correlation of IgG SCO and percent neutralization (top four panels) and IgM SCO and percent neutralization (bottom four panels) data was assessed with simple linear regression (blue dashed line) and 95% confidence bands (grey shading) of the best-fit line. Correlations between IgG SCO, IgM SCO, and percent neutralization were performed on the full sample set PCR-confirmed (N=40), Probables (N=20), Suspects (N=15), and Non-suspects (N=43). Data points are further stratified by day of blood collection since symptoms onset (0 to 65 days) for PCR-confirmed, Probable, and Suspect groups.


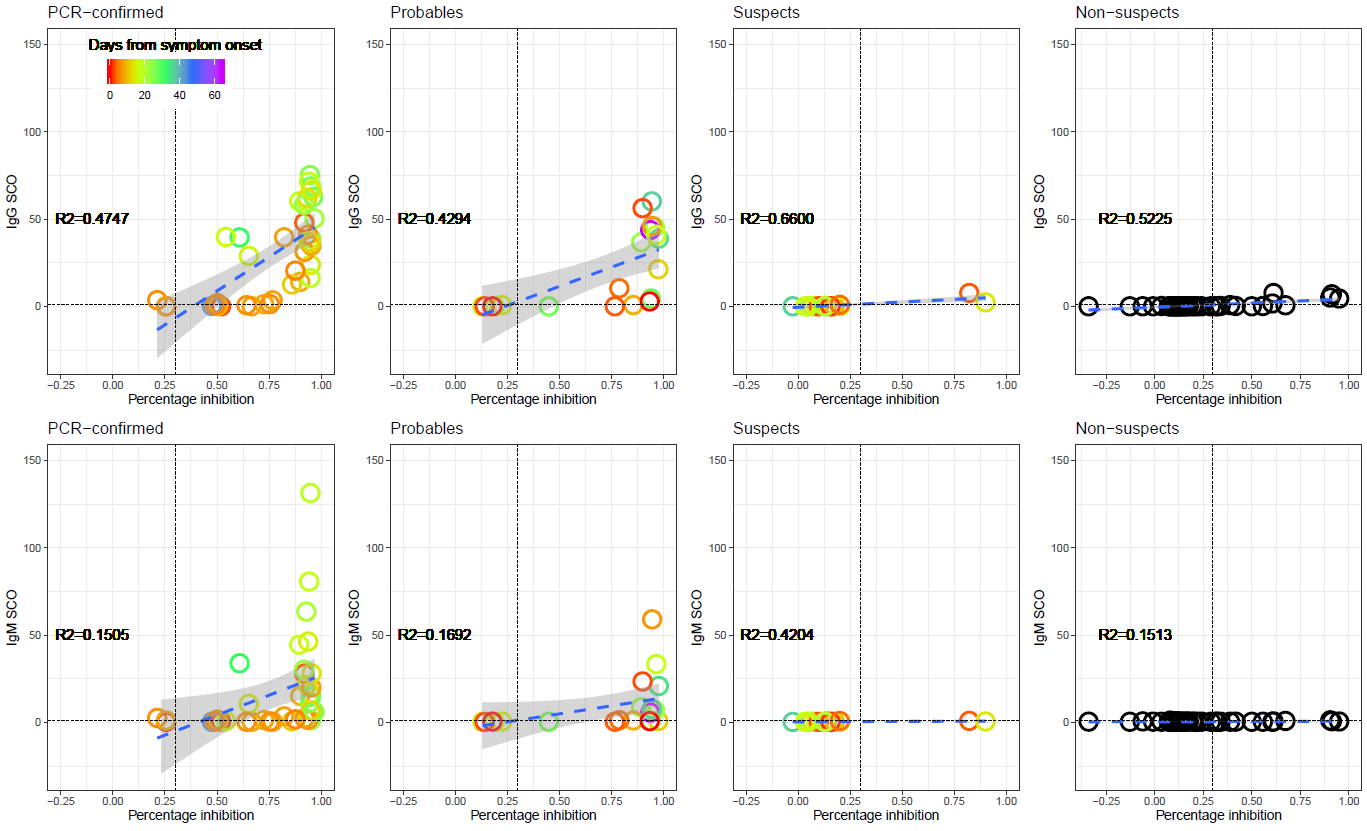


Table S1: Clinical characteristics of PCR-confirmed or Probable cases with high neutralizing antibodies.

|  |  | **PCR confirmed** | **Probables** | **p-value** |
| --- | --- | --- | --- | --- |
|  | N= | 19 | 9 |  |
| **Median (IQR)** | Percent inhibition | 94.9 (93.6-95.1%) | 94.5 (93.9- 96.5) | 0.350 |
|  | Age | 64.0 (60.0-69.5) | 46.0 (39.0- 61.0) | 0.030 |
|  | BMI | 28.9 (24.9-31.6) | 30.9 (26.2- 33.1) | 0.446 |
|  | PCR test from symptom onset | 6.0 (2.5-7.0) | 7.0 (2.0- 28.0) | 0.347 |
|  | Ab test since symptom onset | 14.6 (11.5-22.5) | 23.0 (12.0-35.0) | 0.257 |
|  | IgG SCO | 50.3 (36.9- 62.3) | 40.8 (21.2- 45.4) | 0.090 |
|  | IgM SCO | 17.1 (6.4- 29.1) | 7.1 (0.8- 20.8) | 0.171 |
|  | Length of Hospital stay (in days) | 17.0 (14.0- 21.5) | 18.0 (8.0- 23.0) | 0.740 |
| **Individuals positive**  **N (%)** | Male | 15 (78.9%) | 7 (77.8%) | 1.000 |
|  | **Ethnicity** |  |  | 0.791 |
|  | Black African American | 8 (42.1%) | 3 (33.3%) |  |
|  | Hispanic or Latino | 10 (52.6%) | 6 (66.7%) |  |
|  | White | 0 (0.0%) | 0 (0.0%) |  |
|  | Others | 1 (5.3%) | 0 (0.0%) |  |
|  | **Symptoms** |  |  |  |
|  | Fever | 8 (42.1%) | 3 (33.3%) | 1.000 |
|  | Cough | 8 (42.1%) | 3 (33.3%) | 1.000 |
|  | Shortness of breath | 11 (57.8%) | 6 (66.7%) | 1.000 |
|  | Chills | 5 (26.3%) | 1 (11.1%) | 0.629 |
|  | Diarrhea | 1 (5.3%) | 2 (22.2%) | 0.230 |
|  | Altered mental status | 3 (15.8%) | 0 (0.0%) | 0.530 |
|  | **Chronic medical conditions** |  |  |  |
|  | Hypertension | 14 (73.7%) | 6 (66.7%) | 1.000 |
|  | Heart disease | 3 (15.8%) | 3 (33.3%) | 0.351 |
|  | Diabetes | 12 (63.2%) | 4 (44.4%) | 0.430 |
|  | Liver disease | 2 (10.5%) | 1 (11.1%) | 1.000 |
|  | Lung disease | 4 (21.1%) | 3 (33.3%) | 0.646 |
|  | Kidney disease | 3 (15.8%) | 2 (22.2%) | 1.000 |
|  | **Disease severity at collection** |  |  | 0.408 |
|  | mild | 6 (31.6%) | 5 (55.6%) |  |
|  | severe | 13 (68.4%) | 4 (44.4%) |  |
|  | Hospital outcomes (Recovered) | 17 (89.5%) | 8 (88.9%) | 1.000 |
|  | COVID-19 directed treatment | 16 (84.2%) | 3 (33.3%) | 0.012 |

Table S2: Clinical characteristics of Probables positive or negative of neutralizing antibodies.

|  | **Probables** | **Negative** | **Positive** | **p-value** |
| --- | --- | --- | --- | --- |
|  | N= | 5 | 15 |  |
| **Median (IQR)** | Age | 48.0 (45.0-61.0) | 57.0 (40.0-65.0) | 0.965 |
|  | BMI | 30.7 (26.6-36.0) | 30.9 (25.8-34) | 0.727 |
|  | PCR test from symptom onset | 1.0 (1.0-2.0) | 7.0 (2.0-12.0) | 0.043 |
|  | Ab test since symptom onset | 14.0 (1.9-15.0) | 17.0 (5.5-27.5) | 0.238 |
|  | Length of Hospital stay (in days) | 17.0 (7.0-23.0) | 16.0 (7.5-21) | 0.896 |
| **Individuals positive**  **N (%)** | Male | 3 (60.0%) | 10 (66.7%) | 1.000 |
|  | **Ethnicity** |  |  | 1.000 |
|  | Black African American | 2 (40.0%) | 6 (40.0%) |  |
|  | Hispanic or Latino | 2 (40.0%) | 7 (46.7%) |  |
|  | White | 1 (20.0%) | 2 (13.3%) |  |
|  | **Symptoms** |  |  |  |
|  | Fever | 1 (20.0%) | 5 (33.3%) | 1.000 |
|  | Cough | 0 (0.0%) | 5 (33.3%) | 0.260 |
|  | Shortness of breath | 2 (40.0%) | 11 (73.3%) | 0.290 |
|  | Chills | 0 (0.0%) | 2 (13.3%) | 1.000 |
|  | Diarrhea | 0 (0.0%) | 3 (20%) | 0.539 |
|  | Altered mental status | 2 (40.0%) | 0 (0.0%) | 0.052 |
|  | **Chronic medical conditions** |  |  |  |
|  | Hypertension | 1 (20.0%) | 10 (66.7%) | 0.127 |
|  | Heart disease | 0 (0.0%) | 3 (20%) | 0.540 |
|  | Diabetes | 3 (60.0%) | 4 (26.7%) | 0.290 |
|  | Liver disease | 1 (20.0%) | 2 (13.3%) | 1.000 |
|  | Lung disease | 0 (0.0%) | 6 (40.0%) | 0.260 |
|  | Kidney disease | 0 (0.0%) | 3 (20.0%) | 0.530 |
|  | **Disease severity on admission** |  |  | 0.008 |
|  | Asymptomatics | 1 (20.0%) | 1 (6.7%) |  |
|  | Mild-moderate | 3 (60.0%) | 2 (13.3%) |  |
|  | Hypoxic-No ICU | 0 (0.0%) | 11 (73.3%) |  |
|  | Critical-ICU | 1 (20.0%) | 1 (6.7%) |  |
|  | **Disease severity at collection** |  |  | 0.702 |
|  | Mild-moderate | 4 (80.0%) | 8 (53.3%) |  |
|  | Hypoxic-No ICU | 1 (20.0%) | 6 (40.0%) |  |
|  | Critical-ICU | 0 (0.0%) | 1 (6.7%) |  |
|  | **Disease severity at Peak** |  |  | 0.029 |
|  | Mild-moderate | 3 (60.0%) | 1 (6.7%) |  |
|  | Hypoxic-No ICU | 1 (20.0%) | 11 (73.3%) |  |
|  | Critical-ICU | 1 (20.0%) | 3 (20.0%) |  |
|  | Hospital outcomes (Recovered) | 5 (100.0%) | 14 (93.3%) | 1.000 |
|  | COVID-19 directed treatment | 0 (0.0%) | 7 (46.7%) | 0.008 |

Table S3: Clinical characteristics of seropositive vs seronegative Probables for IgG or IgM

|  | **Probables** | **Seronegative** | **Seropositive** | **p-value** |
| --- | --- | --- | --- | --- |
|  | N= | 7 | 13 |  |
| **Median (IQR)** | Age | 61.0 (46.5- 68.5) | 49.0 (39.0-62.0) | 0.302 |
|  | BMI | 26.6 (25.8- 33.4) | 31.0 (26.2- 34.9) | 0.691 |
|  | PCR test from symptom onset | 2.0 (1.0-2.5) | 7.0 (2.0- 14.0) | 0.175 |
|  | Ab test since symptom onset | 14.0 (2.5- 16.5) | 17.0 (7.0- 28.0) | 0.302 |
|  | Length of Hospital stay (in days) | 17.0 (11.5-24.5) | 13 (7.0-19.0) | 0.606 |
| **Individuals positive**  **N (%)** | Male | 5 (71.4%) | 8 (61.5%) | 1.000 |
|  | **Ethnicity** |  |  | 0.054 |
|  | Black African American | 2 (28.6%) | 6 (46.2) |  |
|  | Hispanic or Latino | 2 (28.6%) | 7 (53.8%) |  |
|  | White | 3 (42.8%) | 0 (0.0%) |  |
|  | **Symptoms** |  |  |  |
|  | Fever | 1 (14.2%) | 5 (38.5%) | 0.354 |
|  | Cough | 0 (0.0%) | 5 (38.5%) | 0.114 |
|  | Shortness of breath | 4 (57.1%) | 9 (69.2%) | 0.651 |
|  | Chills | 0 (0.0%) | 2 (15.4%) | 0.521 |
|  | Diarrhea | 0 (0.0%) | 3 (23.0 %) | 0.5211 |
|  | Altered mental status | 1 (14.2%) | 0 (0.0%) | 0.110 |
|  | **Chronic medical conditions** |  |  |  |
|  | Hypertension | 2 (28.6%) | 9 (69.2%) | 0.160 |
|  | Heart disease | 0 (0.0%) | 3 (23.0 %) | 0.521 |
|  | Diabetes | 3 (42.8%) | 4 (30.7%) | 0.651 |
|  | Liver disease | 1 (14.2%) | 2 (15.4%) | 1.000 |
|  | Lung disease | 1 (14.2%) | 5 (38.5%) | 0.354 |
|  | Kidney disease | 0 (0.0%) | 3 (23.0 %) | 0.521 |
|  | **Disease severity on admission** |  |  | 0.323 |
|  | Asymptomatics | 1 (14.3%) | 1 (7.6%) |  |
|  | Mild-moderate | 1 (14.3%) | 1 (7.6%) |  |
|  | Hypoxic-No ICU | 2 (28.6%) | 9 (69.2%) |  |
|  | Critical-ICU | 3 (42.9%) | 2 (15.4%) |  |
|  | **Disease severity at collection** |  |  | 0.776 |
|  | Mild-moderate | 5 (71.4%) | 7 (53.8%) |  |
|  | Hypoxic-No ICU | 2 (28.6%) | 5 (38.5%) |  |
|  | Critical-ICU | 0 (0%) | 1 (7.7%) |  |
|  | **Disease severity at Peak** |  |  | 0.210 |
|  | Mild-moderate | 1 (14.3%) | 3 (23.0 %) |  |
|  | Hypoxic-No ICU | 3 (42.9%) | 9 (69.2%) |  |
|  | Critical-ICU | 3 (42.9%) | 1 (7.6%) |  |
|  | Hospital outcomes (Recovered) | 7 (100.0%) | 12 (92.3) | 1.000 |
|  | COVID-19 directed treatment | 1 (14.2%) | 6 (46.2%) | 0.328 |

**References:**

1. U.S Food and Drug Administration Emergency Use Authorization. In Vitro Diagnostics EUAs - Serology and Other Adaptive Immune Response Tests for SARS-CoV-2. <https://www.fda.gov/medical-devices/coronavirus-disease-2019-covid-19-emergency-use-authorizations-medical-devices/in-vitro-diagnostics-euas-serology-and-other-adaptive-immune-response-tests-sars-cov-2>

2. Coronavirus (COVID-19) Update: FDA Authorizes First Test that Detects Neutralizing Antibodies from Recent or Prior SARS-CoV-2 Infection <https://www.fda.gov/news-events/press-announcements/coronavirus-covid-19-update-fda-authorizes-first-test-detects-neutralizing-antibodies-recent-or>. 2020.

3. SARS-CoV-2 Neutralization Antibody Detection Kit <https://www.genscript.com/covid-19-news/fda-authorized-genscript-cpass-kit.html>. 2020.
